# Occupy the semantic space! Opening up the language of better regulation

**DOI:** 10.1080/13501763.2023.2181852

**Published:** 2023-03-01

**Authors:** Claudio M. Radaelli

**Affiliations:** a School of Transnational Governance, European University Institute, Florence, Italy; b School of Public Policy, University College London, London, UK

**Keywords:** Advocacy coalitions framework, concepts, ideologies, language, regulation

## Abstract

Policy agendas are often cast in semantic constructions that portray them as universally desirable outcomes. These semantic constructions protect and reinforce the power of dominant coalitions and make it hard to pursue alternatives. The semantic space is entirely occupied by the dominant concepts. At the same time, within the dominant coalition, ideational conflict is muted by decontesting concepts. Drawing on political theory, I show the presence of this double act of reducing the semantic space and decontesting concepts with the case of ‘better regulation’. Then I briefly extend the argument to other terms such as policy coherence, agile governance, smart cities and social value judgements. The critical discussion of the implications of dominant language brings in transparency, allows other coalitions to articulate their vision in a discursive level-playing-field, and offers citizens the possibility to discuss what is really ‘better’ and ‘for whom’.

## Introduction

The motivation for this contribution is to show the practical implications of theory-grounded policy research for the domain of regulatory reform, and extend the claims and arguments to other dimensions of governance. To achieve this aim, I will focus on language. Public policy consists of practices and language. It is language that assigns a meaning to policy practices (Lynggaard, 2019). Indeed, Pierre Muller defines public policy-making as the semantic construction of a relationship between actors and the world in which they operate (Muller, 1995). Policy concepts do not just provide labels describing facts and routines. They are the outcome of the semantic work done by constellations of actors on meanings – my rendition of Muller’s words: *le travail sur le sens effectuè par les acteurs* (Muller, 2005, p. 158). Language is also instrumental in defining interests and institutional missions, and is central to how we think about political order (Cino Pagliarello, 2022, p. 888).

Since Wittgenstein’s proposition (1958) that language is meaning, we know that a single word does not have a unique meaning. Polysemy indicates how a word can have different meanings, variously inter-related. When Holborow (2012) notes that what seems natural in the language about governance, markets, international relations is actually ambiguous, she points to polysemy. Equally, Hannah and Baekkeskov (2020) refer to the polysemic features of the language of ‘One Health’ and Cino Pagliarello (2022) writes about polysemy in the European Union agenda for ‘Europe of Knowledge’. Hence, ambiguity is not an adjective characterizing a certain type of language (like in ‘this is ambiguous language’). Ambiguity is central to how language works in politics.

An elite or dominant coalition in control of a policy domain can carry out political work on polysemy (in Muller’s sense of ‘work done by actors on meanings’) in two directions. First, by stabilizing meanings within those who control the language of a policy, at the same time allowing some internal ambiguity to flow, without disrupting the relationships within limiting the coalition. Béland and Cox (2016) think of polysemy as coalition magnet that attracts different interests and mobilizes otherwise disengaged actors. Second, polysemic political work erects a semantic wall that protects the dominant actors from external criticisms. This is then a double act of keeping cohesion across many different actors with a language sufficiently malleable to accommodate different preferences within a coalition, whilst at the same time protecting the shared semantic space from the (possible or actual) contestation.

Although this is certainly not the first time that language, ideational ambiguity and polysemy are studied in public policy, the implications of the two elements of the double act (how they connect and with what consequences) are not well-known yet. I will show that one way in which this double act proceeds is by presenting the core concepts that make up a policy agenda as if they were anchored to the most naturally desirable outcomes we can think of.

As mentioned, our empirical discussion will revolve for the most part around the so-called ‘better regulation agenda’ in international policy fora. To argue for better regulation seems a naturally desirable agenda, a semantic space where every government and a variety of stakeholders can join in: who wants to say that there is no need to improve on regulation? Who wants ‘worse’ (as opposed to ‘better’) regulation? And yet, behind the generically attractive language of improving and make regulations better, lie a specific set of concepts. Policy concepts define an agenda, are used in dedicated committees of international organizations, shape the choice and transfer of policy instruments, and promote the diffusion of regulatory performance indicators (see the case studies in Dolowitz *et al*., 2020; on better regulation specifically: De Francesco, 2016). Conceptual constructions that look prima facie unquestionable and universally desirable are actually assembled in distinctive ways: they perform the double act of creating consensus within the coalition (limiting internal semantic fragmentation by ‘de-contesting concepts’, as we shall explain below) and narrowing down the sematic space to those who may contest the agenda. All policy concepts are inherently contestable. Policy language – as we have seen above – is polysemic. Indeed, there isn’t a single benchmark to establish the quality of a single rule or a regulatory system (Baldwin *et al*., 2012).

When a dominant coalition performs this double act, what is the semantic space left to those who challenge the dominant coalition and its semantic wall? Panayota Gounari (2006, p. 78) identifies this state of play as closure of meaning: when fixed in the meanings assigned by dominant actors, language presents a reality that is irreversible, natural, fixed and ultimately unchallengeable.

What can policy entrepreneurs or emerging alternative coalitions do in these circumstances? What is the room left for changing language? Following Judith Butler (1992, p. 13), ‘the subject is neither a ground nor a product, but the permanent possibility of a certain re-signifying process’. Social scientists can then contribute to make the ‘permanent possibility’ more likely to happen in two ways: first, by taking what appears as naturally desirable and un-contestable language and de-construct the assemblage of concepts, showing that a given morphology of concepts has its own internal logic and produces choices that are not the only ones possible. Second, re-signifying a given sematic space (such as the space that defines regulatory reform) allows for the possibility of new concepts to emerge, or for concepts to be assembled differently by emerging coalitions. In doing so, social scientists also contribute to transparency in how language is used and for what purposes in public policy processes.

The policy analysis contribution of this exercise in threefold. First comes the central purpose of this Special Issue (SI), which is dedicated to showing the practical implications of policy analysis (Cairney Introduction to SI). Cairney argues that policy process research should aim at bringing to the surface ambiguity and complexity obfuscated by taken-for-granted propositions about public policy. He carries on encouraging realistic expectations for the engagement of policy researchers: to engage with real-world public policies, social scientists should avoid simple images and descriptions of policymaking reality. Instead, they should (re)construct the historical dynamics of policy processes and (de)construct dominant language. This is the same aim pursued here.

The language of governance is inherently polysemic, but is often presented as a ‘given’, a benign ‘big tent’ (Béland & Cox, 2016; Hannah & Baekkeskov, 2020, p. 438) where everyone should sit inside. But there are those outside the tent, and they can be empowered by learning how to de-compose and question the taken-for-granted language.

This article also speaks to ideational policy analysis: here we find a rich and vast literature on discourse, paradigms, ideas and polysemy (Béland, 2009; Cino Pagliarello, 2022; Kamkhaji & Radaelli, 2022; Lynggaard, 2019). By drawing on Freeden on political ideologies and the Rawlsian distinction between concepts and conceptions, we connect the following dots: we see (a) how, inside a dominant coalition, the presence of polysemy (Cino Pagliarello, 2022) does not break down a coalition. Actually, it works in the opposite direction via internal decontestation; (b) how ambiguity gains resistance to critiques by being pinned to general, widely-shared aspirations about how to improve on regulation; and (c) how mechanisms of semantic construction are common in the language of governance. To see this last connection, we will extend the analysis from better regulation to other dimensions of the language of governance.

The third domain is the Advocacy Coalition Framework (ACF) (Pierce *et al*., 2017; Sabatier & Jenkins-Smith, 1993; Weible & Ingold, 2018). Freeden and Rawls – I argue – add clarity to the ACF in terms of belief systems. I do not mean to criticize the ACF but to add depth to the analysis of beliefs in this framework. The scrutiny of better regulation language sheds light on how core belief systems, although based on essentially contestable concepts, become resilient to infra-coalitional contestation internally, whilst presenting externally a uniform front that reduces the semantic space of alternative belief systems. Further, some ACF components can also be justified by political theory – Sabatier's ‘deep normative core’ is an ideology by any other name. Sabatier tends to take the structure of a policy contest as given, with deep normative cores competing through the policy core. What ideological morphology shows is that there is an intellectual process in moving from the deep normative core to the policy core. Freeden’s decontestation highlights this intellectual and political process, and indicates alternative sources of change to those that Sabatier identifies.

Freeden’s political theory explains how dominant coalitions protect core policy beliefs via decontestation. The dominant coalition of regulatory reform portrays the better regulation agenda as identical to the widely shared aspiration towards regulatory improvement, thus limiting the semantic space for alternative coalitions which may exist or may try to emerge. A challenging coalition is discredited by the very fact that it has to disagree with the aspiration to regulatory quality.

Beyond policy analysis, there is also a modest but hopefully not trivial contribution to the critical literature on the so-called neoliberal keywords (Holborow, 2012, 2016). Here I contribute in terms of analysis of morphologies of concepts that dominate policy processes. This literature on neoliberal keywords tends to be macro. It conflates many discourses about policy reforms (such as, in our case, regulatory reforms) into the macro concept of neo-liberalism. By contrast, the analysis presented here is more granular.

Before we start, I wish to make two remarks. First, the critical analysis of language does not prove or deny the achievements of the better regulation agenda, that is, what has been achieved by governments, international organizations like the OECD, the EU and the World Bank. To de-construct a dominant language is an exercise that remains ultimately agnostic about the quality and depth of reforms inspired by better regulation. The appraisal of these reforms across countries is clearly another type of exercise. And in terms of beneficiaries of this article, those who champion better regulation will hopefully benefit too from an understanding of how the language they use does not allow the regulatory conversation to move forward. This article is not for or against a certain actor, neither does it imply the presence of a sort of evil puppeteer orchestrating discourse to suppress alternatives.

Second, when I use ‘dominant’ coalition, I am following the terminology of the ACF – these are objective terms, they do not apply a negative connotation to a coalition that is objectively in charge of a policy sub-system (Sabatier, 1998). And when I talk about the ideological properties of better regulation, I do not mean to imply false consciousness (as explained in the next Section).

In terms of practical organization, the next Section provides the theoretical elements, followed by the discussion of better regulation as political ideology (in the sense of Freeden) across the time period from the 1990s to the present. Empirically, the focus will be on the OECD as key actor in the better regulation agenda (De Francesco & Radaelli, 2023). Having firmed the key analytical points about the semantic mechanisms of better regulation, we will briefly consider how these mechanisms feature in other dimensions of the language of governance. The final Section wraps up the findings, presents the limitations and offers its message for policy practice.

## Theory: conceptual morphology, essential contestability and decontestation

**‘**Better regulation’ has allowed an advocacy coalition to generate waves of adoption of reforms, the consolidation of certain analytical lenses to look at regulation (such as welfare economics), the adoption of a specific set of policy instruments (such as consultation of stakeholders at the early stages of policy formulation, regulatory impact assessment, regulatory and legislative evaluation, and tools for the reduction of administrative burdens) and the diffusion of regulatory oversight institutions (OECD, 2012, 2021a).

What constitutes high quality regulation is definitively a polysemic territory. The space for other coalitions to define alternatives has been limited by a semantic mechanism of identifying a given morphology (the one adopted by the dominant coalition) as universally desirable. Either one works within the ‘big semantic tent’ and therefore cannot contest, or one has to start from the difficult position of being apparently against the universal aspiration to reform and improve.

Potential conflicts within the coalition (for example about the pros and cons of de-regulation) have been limited via another mechanism called, following Freeden, decontestation. The concepts are assembled to protect the normative core (defining what is ‘better’ and what is ‘worse’ regulation) whilst allowing for a certain level of flexibility within the ‘big tent’. Together, the two mechanisms create the double act that reduces the semantic space for alternative discourses.

Political theory offers precious insights on the double act. At the outset, we must embrace a specific position on ideology. In common parlance, ideologies are often seen as beliefs that provide a distorted, power-seeking framework to mobilize social action and justify certain political institutions or political visions. Their illusory nature is central to Marxist critiques of ideology as false consciousness (Freeden, 1996, p. 1). When we say that someone is an ideologue, we attribute negative elements to ideological thought. Since, as mentioned, I do not appraise the quality of better regulation reforms, such a loaded understanding of ideologies is not appropriate here. Instead, Freeden has a non-normative starting point. For him, political ideologies are clusters of distinctively arranged concepts – arranged in such a manner that the key ideational constructs (say, freedom, equality and justice) support each other and make a coherent whole. The specific arrangement of ideational constructs in a given time and place is what Freeden defines a morphology of concepts.

These conceptual interlinkages (that is, the morphology) make up an understanding of the political world that actors occupy, as well as providing the possibility to act (on the basis of that understanding). Ideologies ‘guide practical political conduct’ (Freeden, 1996, p. 6). Freeden adds (1996, p. 4) that ‘the nature of society and its structures, supposedly *reflected* in ideologies, are themselves partly the *product* of those ideologies, operating as ways of organizing social reality’ (emphasis in original). Ideologies, then, are more than lenses on, and interpretations of, society.

Further, morphological patterns should not be studied exclusively in terms of thought-processes and their logical relations. They can be approached empirically, by observing their cultural representations, historical instances, and the institutions and fora where they emerge and consolidate. Ideologies become a set of concepts leveraged to act on the world through the repeated interaction of actors (that is, practices) within specific institutional bodies or social fora. In these bodies and fora ‘the conventions through which [an ideology] is understood and perceived’ (Freeden, 1996, p. 54) solidify. Examples in the international domain of better regulation are the Regulatory Policy Committee of the OECD, the Competitiveness Council of the European Union and networks like the Directors and Experts of Better Regulation of the European Commission (Radaelli, 2020, 2021; Radaelli & De Francesco, 2007). Back to Freeden, ideologies make up the thought-behaviour that leads to the identification of the practices – indeed, ideologies are referents (this is the term used by Freeden) with a dual practice/thought-behaviour function.

So far, we have considered ideologies as morphologies of concepts. But, are these concepts immune from critique? How does an ideology protect its concepts? A given, existing morphology is just a configuration drawn from a pool of ‘indeterminate and unlimited combinations. That indeterminate range is the product of the essential contestability of political concepts, and essential contestability provides the manifold flexibility out of which ideological families and their subvariants are constructed.’ (Freeden, 1996, p. 4). From this follows the essential contestability of a morphology (Freeden, 1996, p. 55; see also Walter Bryce Gallie’s seminal contribution, Gallie 1955–1956). Essential contestability of an ideology comes from the fact that the morphology of concepts provides a sort of topography of a political reality. This topography is built around preferences on the range of components to be included, as well as the indeterminacy of the components included (Freeden, 1996, p. 57). Freeden provides the example of equality – a concept that is then broken down into components of ‘equality of opportunity’ and further down decisions about the exact meaning of ‘equality of opportunity’ in relation to specific policy choices.

In our case, better regulation must be broken down into components, otherwise it could not lead to any practice or any regulatory reform. This breaking-down step is well-illustrated by John Rawls. Drawing on Hart (1994/1961), in his *Theory of Justice*, Rawls (1999/1971, p. 5) distinguishes concept and conceptions: ‘it seems natural to think of the concept of justice as distinct from the various conceptions of justice and as being specified by the role which these different sets of principles, these different conceptions, have in common’. Justice is intuitively understood by all as desirable, a general desire, a value. Conceptions of justice may vary. The level of agreement on these conceptions also varies across societies and in time.

Whether, in a given policy reform agenda, contestability (in the sense of Freeden) and the measure of agreement on conceptions (Rawls) is openly unveiled and accepted, or denied, is an empirical question concerning coalitions active in a policy process where the agenda emerges and consolidates across the years. The ACF is the appropriate lens on the policy process to examine this.

Turning then to the ACF, policy subsystems (better regulation being the policy subsystem in our case) should be studied over a period of a decade or more, to examine how coalitional dynamics, learning and change unfold. Within this time-frame, we observe at least one coalition (which is by default dominant in the subsystem) or two or more coalitions in competition. A coalition can only emerge when there are individuals with a common belief system (Sabatier & Jenkins-Smith, 1993). Actors of the same coalition tend to share beliefs about the attributes of a problem, the appropriateness of policy instruments, and the aim of a policy (Sabatier & Jenkins-Smith, 1993; Weible & Ingold, 2018).

Beliefs include perceptions and causal understandings of a policy problem, as well as relative priorities of values (Sabatier & Jenkins-Smith, 1993). In turn, belief systems are organized in hierarchical ways. The deep core beliefs are fundamental normative axioms about the nature of the individual, or the role of the state in social and economic activity. Going down the hierarchy, we find beliefs associated with public policy: they are core policy beliefs and secondary beliefs. The policy core beliefs of a coalition maintain cohesiveness over time, while secondary beliefs are narrower and focused – they can differ within an advocacy coalition.

Let us now assemble political theory and the insights of the ACF. Freeden and Rawls talk about ideologies, concepts and beliefs as political theorists, but their approach can travel into ACF language. Freeden identifies an ideology as the morphology of concepts, Rawls would call the concepts assembled in an ideology ‘conceptions’. Rawls would indeed say that better regulation is a concept, whilst beliefs orienting the choice of policy instruments and regulatory practice are conceptions. Conceptions vary within a coalition: this is a classic case at the international level, where delegates in OECD bodies and the EU represent different governments over time, experts may change their opinions on risk and regulation, and external shocks may orient a regulator towards precautionary beliefs, another towards risk.

The variability of conceptions puts the cohesiveness of a coalition at risk. But here is where Freeden’s approach sheds important light. Political ideologies shield infra-coalition contestability. They do so by presenting the ideology as a configuration of decontested meanings of concepts. Listening one more time to Freeden (1996, p. 76):

In concrete terms, an ideology will link together a particular conception of human nature, a particular conception of social structure, of justice, of liberty, of authority, etc. ‘This is what liberty means, and that is what justice means’, it asserts. Ideologies need, after all, to straddle the worlds of political thought and political action, for one of their central functions is to link the two. The political sphere is primarily characterized by decision-making, and decision-making is an important form of decontesting a range of potential alternatives. Thus, while the very nature of political concepts lies in their essential contestability, the very nature of the political process is to arrive at binding decisions that determine the priority of one course of action over another.

Decontestation refers to how Rawlsian conceptions are portrayed within a coalition. An ideology seeks to decontest its own conceptions and make them acceptable within the overall framework. But of course, at the same time, a dominant coalition is involved in discrediting the meanings of the same conceptions within other ideological frameworks – like liberals discrediting how the concept of freedom is understood by libertarians. In policy research, Cino Pagliarello (2022) has shown how polysemic governance makes it possible to carry out reforms over relatively long-periods of time, even in the presence of clearly observable ambiguities.

Now we have the key elements needed for our analysis. The emphasis on concepts and their morphology means that we do not need to directly examine narratives, myths and idioms. These are additional dimensions and empirical details of an ideology. The approach is parsimonious and powerful at the same time.

## Better regulation

Morphologies of concepts are referents that constitute (part of) reality and guide practice. Political ideologies are anchored to desirable concepts, such as equality and justice. However, their conceptions are inherently contestable. By decontesting alternatives, ideologies protect their normative core and allow constellations of actors to achieve decisions on specific cases. The element of essential contestability is obscured, unveiled so to speak.

To study ideologies in the sense of Freeden, we must consider morphologies of concepts in a specific space and time. In our case, the ‘space’ is populated by international organizations (mostly the OECD, but also the World Bank and the European Union, see Radaelli, 2021) that have supported the emergence and diffusion of the better regulation agenda. As for time, we need a sufficiently long period of time to examine how the morphology came about in that ‘space’. Let us then turn to a brief historical account.

The better regulation agenda has its origin in the experience of countries like the United States (regulatory impact analysis of proposed regulations was introduced in 1981 by Ronald Reagan with Executive Order 12291) and the UK (a country that already in 1985 mandated compliance cost assessment to quantify the impact of new regulations on business, in response to political apprehensions about over-regulation). But it was in the 1990s that better regulation as policy agenda emerged in the OECD – the pivotal organization because of its ideational power-force and its dedicated fora on regulatory reform (Radaelli, 2020).

The first OECD attempts to codify better regulation originated in PUMA (Public Management Committee) in 1995. PUMA was the OECD body dedicated to public management and public sector reforms – thus establishing, at that time, a connection between regulatory reform and the new public management (for a discussion of this connection see Radaelli & Meuwese, 2009). In the 1990s, PUMA was led by Scott Jacobs, who brought to the OECD his knowledge of regulatory analysis as carried out in the US federal government.^1^ International organizations, especially the World Bank and the European Commission, became active on better regulation in the same period.^2^

PUMA prepared the ground for the 1995 Recommendation of the OECD Ministerial Council on Regulatory Reform – effectively the first morphology of concepts, delineating the contour of better regulation. Regulatory reform was described as a response to ‘troublesome problems’ arising because of the ‘maturing and expansion of regulatory systems’ (OECD, 1995, p. 8). The three central conceptual elements were: the need to manage and when necessary tame regulatory growth, in terms of regulatory costs and quantity of rules; the design of standards and benchmarks of high quality regulation; and, finally, legitimacy: ‘Throughout the OECD area, administrative openness and responsiveness have become more important’ (OECD, 1995, p. 8). In the name of better regulation, these three dimensions muted (or decontested, in Freeden’s language) a possible clash between an explicit de-regulatory trajectory and another agenda, more concerned with quality and transparency. In other words, both countries with de-regulatory executives and countries less attracted by de-regulation found home in this Recommendation.

The overall tone of the Recommendation was however quite explicit on the problem created by increasing regulatory costs and over-regulation. Further, this morphology was glued (and still is today) by a belief in economics as dominant discipline to understand, measure and manage regulation. As shown by Morgan (2003), with better regulation the framework to judge the quality of rules is, broadly speaking, grounded in economics: costs and benefits – she argued – matter more than values. This shapes how bureaucrats frame regulation in terms of net benefits and mutes the possibility to make the case for regulation by leveraging values. It’s all about how regulators justify new interventions: cost–benefit ratios or values? Indeed, the subtitle of her book is *The Bureaucratic Politics of Regulatory Justification*.

Back to the OECD now. To respond to concerns and expectations about regulation, the OECD Council introduced a checklist for Regulatory Decision-Making (OECD, 1995, pp. 10–14). This checklist was *grosso modo* modelled around regulatory impact assessment (RIA) as known in what was the international experience at that time. RIA became the pivotal instrument in better regulation. Inside the 1995 RIA-inspired checklist, we find regulatory issues framed in economics (as argued by Morgan, 2003). New regulations – the OECD checklist states – need to pass the tests of whether: the policy problem is correctly defined; government action justified (recall Morgan’s book subtitle about justification); regulation is really the best form of government action; and, in question 6 of the checklist, possibly the most explicitly economics-oriented, whether the benefits of proposed rules justify (one more time we find the verb ‘to justify’) the costs.

To support the Recommendation, PUMA published a document on best practice in RIA and thematic studies on regulatory reform (OECD, 1997a, 1997b). The studies argued for reforms acting decisively on burdens, compliance costs and transparency improve market entry and regulatory growth. As for citizens, the OECD argued that less paperwork would free up individual initiative and precious time. Alongside the thematic studies, the ‘best practice’ volume (OECD, 1997a) pinned down the concept of better regulation to a policy instrument, RIA. Governments were consequently invited to adopt checklists with RIA features to manage their regulations.

As shown by De Francesco (2012), the OECD, 1995 Recommendation was the origin of a diffusion wave across the OECD countries. The World Bank accompanied diffusion in developing countries. The European Commission embraced better regulation a bit later, in the early 2000s (Radaelli, 2020; Radaelli & De Francesco, 2007), but since then it has amplified diffusion by making better regulation an element of the discussions with prospective members of the European Union. The Member States of the EU endorsed better regulation and the pivotal role of RIA in the Mandelkern Report (2001).

In 2012, the OECD published a second Recommendation (OECD, 2012), yet again highlighting the role of RIA as a fundamental policy instrument, this time with the ambition of deploying evidence-based tools throughout the life-cycle of regulations and through the whole government – from risk assessment to periodic reviews of legislation, systematic engagement of stakeholders, judicial review and co-ordination mechanisms between levels of government. On 6 October 2021, the OECD Council adopted a Recommendation on Agile Regulatory Governance to Harness Innovation (OECD, 2021b).

With this recent Recommendation, the reach of better regulation extends to innovation. In Rawlsian terms, this means a concept gathering an increasing semantic space in terms of conceptions. Following Freeden, the morphology of concepts becomes more encompassing. One more time, in the 2021 Recommendation we find the emphasis on appraisal instruments (including horizon scanning and scenario analysis to enhance RIA) and regulatory assessment cycles, with the goal of creating and managing regulation that are ‘fit for the future’ and ‘help innovators navigate the regulatory environment’ (OECD, 2021b, p. 4).

Recall that a property of political ideologies as described by Freeden is their capability to address a range of options about how to interpret concepts and transform them into action without breaking up the ideology. This is done, first, via decontestation of concepts that are actually essentially contestable. But second, and adding to Freeden’s framework, the mechanism is more likely to be triggered and persist over time if there is stability at the level of fora and key individuals. Since the 1995 Recommendation, the OECD has provided the forum for Ministers and high-level experts to elaborate on how better regulation must be implemented (first within PUMA, later in the Regulatory Policy Committee). The presence of Nikolai Malyshev at the top of the Regulatory Policy Division for 12 years provided stability to the OECD forum. The leadership of Gary Banks as chair of the Regulatory Policy Committee (as well as chair of the Productivity Commission in Australia) created a formidable impulse to this Committee (Radaelli, 2020 for more details). The European Commission established in the early 2000s a group of Director and Experts of Better Regulation that still exists today (Radaelli and De Francesco, 2007). The World Bank set up communities of learning and, in the second half of the 2000s, the Better Regulation for Growth Programme,^3^ to consolidate the network of regulatory reformers in developing countries – always with RIA as central policy instrument.

Whilst the dominant coalition found the fora to meet up, consolidate network-type relations, and engage with the development of better regulation, the reality on the ground has always been quite diverse. In the EU for example, better regulation conceptions were refracted by bureaucratic traditions and culture, the capacity of the executive to absorb and govern conflict, the features of the law-making process, and the make-up of the constellation of actors involved in implementation (these were the variables identified in that period, see Radaelli, 2005; other variables at work in implementation were empirically detected a few years later by De Francesco *et al*., 2012). Yet there were no signs of governmental actors breaking up with the better regulation agenda. For example, in the 2000s the Netherlands and the UK wanted to establish targets for the reduction of administrative burdens, whilst the European Commission preferred a wider system of regulatory indicators (Radaelli, 2020). These days the Council and the European Commission do not have the same position on the innovation principle (Taffoni, 2020). The OECD is engaged in a better regulation 2.0 project, showing that this organization is aware of the need to raise questions about the vision and the tools of this reform agenda. An OECD working paper maps the structure of belief systems within the OECD’s Regulatory Policy Committee and experts, showing, with the aid of Q methodology, a differentiated landscape of beliefs (Radaelli *et al*., 2022). Yet the overall agenda has never been challenged by defections – the OECD Member States are still very much together under the semantic banner of better regulation – evidence that decontestation inside the dominant coalition has worked effectively, and for a long time.

## The troubled attempts to opening up the semantic space

There are different ways to open up the semantic space. Essentially, they are all attempts to shed light on the difference between concept and conceptions, allowing ‘alternatives ways of organizing social reality’ (Freeden, 1996, p. 4). Some are supported by emerging coalitions, others are academic attempts, some others come from within the experience of governments. Some are meant to lead to explicit contestation, others to more transparency and awareness in the dominant coalition.

Let us start with attempts to contest by arguing that regulatory reform agendas are ‘better’ for some but nor for others. In the EU, the European Trade Union Institute (ETUI) has argued against the conceptions implied by the better regulation language. For ETUI, the narrow cost-oriented practice of impact assessment in health and safety (narrow in the sense of not giving an adequate attention to long-term benefits) and the initiatives to ‘modernize’ the *Acquis Communautaire* by reducing administrative obligations are attacks on the European Social Model (Schömann, 2015; Van den Abeele, 2014, 2015). In 2015, these concerns materialized into an umbrella organization called ‘better regulation watchdog’^4^ supported by 66 green, social and consumerist groups across the EU. Corporate Europe Observatory labelled better regulation ‘corporate-friendly deregulation in disguise’.^5^ Friends of the Earth Brussels commissioned cartoons to Ralph Underhill^6^ in the same period (2015–2016). On the academic side, Garben and Govaere (2018) edited a collection of critical contributions. In her conclusions, Garben claims that better regulation can aggravate the de-regulatory trajectory of the EU, with negative implications for social rights and consumer, environmental and health protection (Garben, 2018, p. 241).

These efforts, however, have not cemented into a new morphology of concepts due to the difficulty of forming a stable semantic space alternative to better regulation – and an advocacy coalition grounded on these alternative beliefs. To say that EU better regulation is flawed does not shed much light on what the ‘true’ better regulation looks like. In other words, the actors that argue against EU better regulation have not produced an alternative morphology of concepts, since their initiatives have been limited to unveiling the negative features of better regulation. In Europe, the best days of a possible alternative advocacy coalition were in the mid-2010s – the political momentum for alternative morphologies of concepts seems to have faded since then.

In the USA, experts and academics have taken the lead in exposing the negative implications of better regulation. In that country, the efforts have been directed towards a reformulation of the morphology of concepts underlying specifically benefit–cost analysis rather than the broader better regulation ideology. This is because in the USA benefit–cost analysis is the central concept in all executive orders issued by successive Presidents on regulatory scrutiny and oversight.

The Centre for Progressive Reform^7^ gathers more than 60 scholars – many with expertise in regulation. Although not partisan, Resources for the Future,^8^ the George Washington Regulatory Studies Center^9^ and the Penn Programme on Regulation^10^ have criticized the de-regulatory zeal of the Trump administration, showing among other things that Trump did achieve much less than he claimed (Coglianese, Sarin, & Shapiro, 2021). University lawyers like Doug Kysar (2010) have argued that benefit–cost analysis does not stand up to scrutiny from within its own foundations. An economic analysis showing that the elimination of humans would produce benefits to biodiversity and the survival of the planet would lead to the conclusion that we should plan the elimination of the human race. Benefit–cost analysis of pollution does not take into consideration the disappearance of species of fish that are not consumed – because these species have no market value (Kysar, 2009). Absent normative foundations, we are regulating ‘from nowhere’, that is, without any normative, reasonable compass.

Other university researchers have shown the way to reform and re-think benefit–cost analysis in light of sustainability, environmental protection and climate policy. Livermore and Revesz^11^ (2020) have put forward the argument that we should not apply the discount rate to benefits that occur to the next generations, because this would be the same as saying that the benefits of our children count less than our own benefits – thus violating basic notions of equality and inter-generational fairness. Other important work has exposed how administrative burdens limit the rights of citizens and create more inequality (Herd & Moynihan, 2018). The debate in the USA is therefore lively, but focused on the reforms introduced by US administrations. It does not reach out to the broader comparative scene and international coalition supporting better regulation in international organizations.

Let us now look at attempts to provide discursive evolution within the dominant coalition. A possible evolution, centred on regulatory stewardship, emerged in New Zealand, from within the government, in particular Treasury and the Ministry of Business, Innovation and Employment (MBIE) (Ayto, 2014; Van der Heijden, 2021). In this case, the morphology of concepts proceeds from the core belief that regulatory frameworks are assets. An asset is something that over time is expected to generate a flow of benefits. Since regulations are expected to produce net benefits over time, regulatory arrangements should be considered assets. This stands in contrast to regulation as liability, a potential obstacle to business activity, a cause of corruption, the origin of red tape and other negative imageries.

The next step in this morphology of concepts is to connect regulation-as-asset to the concept of stewardship. If regulation is a valuable asset, departments should be charged with stewardship responsibility, embracing a comprehensive, long-term duty of care for a resource that exists for the benefits of the whole society. The morphology extends to the approach to regulations. Rather than limiting regulatory appraisal to examining regulations one by one, with ex ante impact assessment (RIA) or ex post regulatory evaluation, the stewardship approach provides a focus on the whole system – for example set of rules and laws affecting a whole sector like climate.

Departments then become precious custodians, with tasks of advising elected politicians, performing horizon scanning exercises, carrying out proactive periodic maintenance (independently of the level of political attention for that), and promoting long-term resilience of their regulatory assets. Importantly, this approach does not recognize value to blunt de-regulatory approaches, such as one-in-x-out targets that force departments to eliminate costs in a given sector when new regulations introduce new costs – the notion of X means that one can have one-in-one-out or one-in-two (or even three)-out (on these targets, see Trnka & Thuerer, 2019).

In New Zealand, stewardship is a statutory responsibility for central government departments since 2013 (State Sector Act 1988, Section 32, as amended in 2013 to include stewardship of legislation), although the legislation is exhortatory (there are no penalties or sanctions). Stewardship entails a precise set of expectations, originally set in 2013 and then updated in 2017,^12^ about monitoring and review of existing legislation; analysis and implementation support for changes to regulatory systems; and good regulatory practice. Since 2020 New Zealand has a Public Service Act^13^ – the 1988 State Sector Act was replaced by this new Act – and a Public Service Leader for ‘regulatory system stewardship and assurance’ appointed by the Head of the Public Service. The approach is centred on the bureaucracies – because one cannot realistically expect elected politicians to perform these duties. If anything, politicians have a tendency to set new rules and ‘forget’ about their implementation, maintenance and evaluation. By contrast, the key drive of the public service is supposed to be resiliency. Within regulatory stewardship, resiliency comes before efficiency.

Regulatory stewardship does not allude to something generally considered desirable (like better regulation). It does not ‘occupy’ all the semantic space, making alternatives sound impossible. Conceptually, it has a connection with the academic literature on regulation. Indeed, stewardship resonates with Majone’s concept of regulatory agencies and bureaucracies in general as trustees. Majone (2001), when criticizing the mode of delegation presupposed by principal agent-models, argued that independent regulators are better understood in a mode of trusteeship, based on fiduciary relations and the concept of trust.

At the moment, the discursive turn of New Zealand has not spread throughout the world. Neither can we appraise how deep and grounded in daily practices this turn is in New Zealand, without specific empirical research on the connections between this emerging language and the practice of regulation in that country. But some principles endorsed by the European Commission such as ‘evaluate first’ resonate with this morphology of concepts – unsurprisingly, this comes from a bureaucracy, the Commission, relatively insulated from elected politicians because of its right to initiate legislation and the special ‘duty of care’ the Brussels executive has in relation to the body of EU legislation. The OECD Regulatory Policy Committee has explored the extension of the experiment carried out in New Zealand to other members of the organization with a dedicated seminar on 11 May 2022. Whether this will extend to a reformulation of the morphology of concepts in the dominant coalition is clearly too early to tell.

## Extending the argument

We found the following semantic features of better regulation: a morphology of concepts that contains different conceptions, the decontestation of contradictions and inconsistencies within the dominant coalition, and the silencing of alternatives. Although we cannot re-construct these features for other key-words dominating the language of governance in international organizations (Grindle, 2007), we can nevertheless illustrate other domains where the semantic space is close, hard to challenge and to open up. In essence, these semantic features appear in the adjective that qualifies our shared and intuitive notion of good governance. Going back to Rawls, good governance is the concept, the adjectives (such as coherent, agile and smart) are the conceptions.

One is policy coherence. Among international institutions, the OECD and the United Nations are particularly active in promoting it. The OECD adopted in 2010 and revised in 2019 a Recommendation of the Council to promote policy coherence for sustainable development (OECD, 2019). The UN Committee of Experts in Public Administration (CEPA) recently developed a set of principles to address the governance challenge of implementing the 2030 agenda. In 2021, the Department of Social and Economic Affairs published a guidance note on policy coherence for governments and public officers.^14^ The guidance note observes that

Achieving, or making progress on, one target can either boost progress on another target (“synergy”) or make it more difficult to achieve another target (“trade-off”). Recognizing these interdependencies and interactions– “the integrated nature of the SDGs”, as the 2030 Agenda preamble puts it–is a key first step to ensure that public policies are coherent with one another and will achieve their intended results. (Nilsson, 2021, p. 2)

The document carries on with the claim that ‘The absence of coherence may result in many types of governance problems, such as compartmentalization, fragmentation, competing and incoherent objectives, and inconsistent policy mixes’ (Nilsson, 2021, p. 2).

In short: bad things may happen if policy-makers are not coherent. Who can be against policy coherence? Who can possibly argue for incoherence? And yet, long time ago, Grant Jordan and Darren Halpin (2006) exposed the incoherence of policy coherence. Coherence must meet the conditions for rational comprehensive policy-making – there has to be a planner who has sufficient knowledge, information and computational ability to decide how to assemble the policy silos and ease trade-offs across policy domains and departments. Since these conditions are not met in governance, it is intellectually impossible^15^ to achieve objective agreement in terms of how coherence is and how it should be achieved. When we observe ‘incoherence’ we are actually in front of partisan mutual adjustment, diverse and legitimate objectives, different positions that increase the level of information brought to a policy problem (Jordan & Halpin, 2006; drawing on Lindblom, 1979). Further, politically speaking, the constituencies for two policy domains or silos can be powerful, whilst the constituency or coalition for the merged ‘coherent’ policy sector may be absent. The level of fragmentation in governance is a function of interest group and coalitional mobilization.

Since in the real world the conditions for synoptically rational policy-making and the perfect alignment of pressure groups are absent, and, instead, bounded rationality is common, it is incoherent to argue for coherence. Jordan and Halpin conclude: ‘Competition and conflict are the essence of politics: to deny their existence is unrealistic. The irony is that politicians seem unhappy with policymaking processes and models that accept the political nature of decision-making’ (Jordan & Halpin, 2006, p. 39).

Other examples are agile governance, smart cities and value-for-money. Who can stand up and make the case for lethargic governance, dull cities and object to value-for-money? Agile governance was championed by the Finnish agency Sitra already in 2014 (Doz & Kosonen, 2014), and developed by Luna *et al*. (2020). A comprehensive report of the OECD Observatory of Public Sector Innovation deals with a whole set of ‘governance with adjectives’,^16^ such as anticipatory, reflexive, adaptive, experimentalist, tentative and, of course, ‘agile’ (Tõnurist & Hanson, 2020, p. 35).

However, prudent and humble governance can be more realistic and superior to agile, especially if agile governance demands the skills of a public sector super-bureaucrat or super-hero. Interestingly, the government of Finland has promoted a reflection on humble government which draws on Charles Sabel’s approach to governance. Humility is linked to continuous learning and a conception of governance as experimentation in a world of uncertainty, where many different stakeholders possess information about how to solve problems (Annala *et al*., 2020).

The language of smart cities puts a positive semantic veil on transformation projects that are lucrative for certain economic actors, but not without important winners and losers in society (Michalec *et al*., 2019 on the case of Bristol, UK). Value-for-money can obfuscate other important values in public service provision. In the UK, the notion of ‘best value-for-money’ has been foundational to the operations of the National Institute for Care and Clinical Excellence (NICE), the body that sets the priorities for primary health-care. NICE argues that, in setting priorities, it follows social value judgements. Yet the morphology of concepts behind social value judgements reveals an anchorage to value-for-money and a specific (and contestable) school of welfare economics. As demonstrated by Charlton and Weale (2021), social value judgements is a language that does not allow NICE to articulate with the necessary precision the basis for its decisions:

the notion of the ‘social value judgement’ has proved insufficient to the task of adequately describing and justifying the normative basis for decision-making and may, in fact, have created a barrier to clearly conceptualising NICE’s evolving approach and ensuring its continuing coherence. (Charlton & Weale, 2021, p. 508)

Charlton and Weale suggest the notion of practical public reason as alternative foundation of the language to set priorities.

In conclusion, better regulation is not a single case. The language of governance provides other policy agenda scripts where polysemy is exploited by dominant coalitions via the double act of creating internal semantic cohesion and silencing alternatives. We have seen cases where the intellectual foundations are shaky – see ‘the incoherence of policy coherence’ and ‘value-for-money’ – nevertheless dominant concepts are presented as obvious, natural and desirable.

The presence of the double act mechanism in so many sectors indicates a general pathway to policy entrepreneurs who want to contest the big tents of dominant language: show the fragility of intellectual foundations, expose internal ambiguity camouflaged by decontestation, gain a discursive level-playing-field, re-configure polysemy in ways that are more transparent and inclusive (as in the case of ‘regulatory stewardship’).

## Conclusions

By leveraging Freeden’s approach to political ideologies, Rawls’s distinction between concepts and conceptions, and by integrating the ACF, this article has shed light on the semantic politics of regulatory governance. To approach the language of governance as a morphology of concepts is useful to capture the difference between better regulation and regulatory stewardship – hence our approach is also useful to explore discursive evolution within a policy domain and to appraise alternative semantic constructions. Better regulation is not the only domain where these semantic mechanisms operate: the language of governance and policy reforms offers other examples, as we have seen. Some of these morphologies of concepts of governance obfuscate winners and losers (as mentioned in the case of smart cities), others do not provide the correct basis for taking decisions (such as social value judgements). In the case of policy coherence, there are good analytic reasons to argue that we should not necessarily consider it a valuable goal in a world of bounded rationality.

For ACF theorists, the take-away messages of this article are about the ideology-like structure of belief systems in advocacy coalitions, the mitigation of infra-coalitional conflicts (via decontestation), and the semantic mechanisms that silence alternative coalitions. Freeden and Rawls describe the intellectual process going from the deep normative core to the policy core.

The contribution to the literature on discourse (Lynggaard, 2019), ideas (Béland, 2009; Béland & Cox, 2016), polysemy in public policy (Cino Pagliarello, 2022) and critical discourse analysis (Fairclough, 1995) is to show how to de-construct a particular form of dominant language (based on the double act we described) and to open up the semantic space. Ambiguity does not necessarily break-up the dominant coalition, it can be exploited to build up a big semantic tent.

Further, the approach presented here is granular and does not conflate the presence of ambiguity in dominant discourses with the category of neo-liberalism – a label that does not help if it conflates too many rhetorical and semantic registers of justification of policy change and reforms.

These results come with limitations: as mentioned, this approach is unable to appraise the quality and depth of cross-country regulatory reforms inspired by the better regulation agenda; the analysis of discourse must be supplemented by an analysis of practices that only in-depth observation of OECD and other fora allows (Radaelli, 2020, 2021); and finally, the double act we described is only an element of the coalitional competition described by the ACF.

In terms of policy practice, to understand how these mechanisms of language work brings in transparency and allows a more diverse dialogue about the advantages and limitations of regulatory reforms, without obfuscating what is being done under generically attractive labels. Further, providers of public management executive training should be able to discuss the tools they teach by opening up their semantic horizon, considering terms like regulatory stewardship that allow for an open discussion of regulatory ethics and accountability with practitioners.

Looking critically into the language that policy-makers take for granted is a valuable task that can be carried out by engaged policy scholars and policy entrepreneurs. Unveiling and exposing the double act can empower alternative coalitions but also benefit the members of the dominant coalition willing to reduce ambiguity and increase transparency in the connection between their language and their practice. In the end, all concepts are contestable: policy researchers can contribute to keep this important door (to contestation) open. The identification and critical discussion of dominant language brings in transparency in the policy process, allows other coalitions and policy entrepreneurs to articulate their vision, and offers citizens the possibility to discuss what is really ‘better’ and ‘for whom’.

## Notes

1 Jacobs worked at the US Office for Management and Budget between 1985 and 1991. From 1995 until January 2001 he ideated and directed the OECD programme on regulatory reform within PUMA. Source: http://regulatoryreform.com/wp-content/uploads/2014/09/Scott_Jacobs_CV_JCA_Sept_2014.pdf.

2 Interestingly, the World Bank Governance Practice that championed better regulation and developed the Better Regulation for Growth Programme has been led since 2005 by Peter Ladegaard, previously at PUMA with Scott Jacobs. Source: https://www.worldbank.org/en/about/people/p/peter-ladegaard On the European Commission / EU scene, see the literature review by Listorti et al. (2019).

3 Information on this programme, including the final report, is found at https://openknowledge.worldbank.org/handle/10986/27866.

4 https://www.foeeurope.org/sites/default/files/other/2015/brwn_founding_statement_and_members.pdf.

5 https://corporateeurope.org/en/better-regulation-corporate-friendly-deregulation-disguise.

6 https://markavery.info/2016/04/30/saturday-cartoon-ralph-underhill-14-9/.

7 http://progressivereform.org.

8 https://www.rff.org.

9 https://regulatorystudies.columbian.gwu.edu.

10 https://www.pennreg.org.

11 Interestingly, on 2 September 2022 the US President Biden nominated Revesz to be Administrator of the Office for Information and Regulatory Affairs.

12 See https://www.treasury.govt.nz/publications/guide/government-expectations-good-regulatory-practice.

13 See https://www.legislation.govt.nz/act/public/2020/0040/latest/LMS106159.html Section 12 covers the public service principles: political neutrality, free and frank advice, merit-based principles, open government, and stewardship. In turn, stewardship is defined to include among other dimensions “legislation administered by agencies.”

14 The note was authored by Måns Nilsson, Executive Director of the Stockholm Environment Institute. Here we reference this UN document as Nilsson (2021).

15 Meaning that synoptic rationality cannot be achieved.

16 I am grateful to Lorenzo Mascioli for this expression, who reminds us of the debate on ‘democracy with adjectives’ (Collier & Levitsky, 1997).
